# ChildTalks+: a study protocol of a pre-post controlled, paired design study on the use of preventive intervention for children of parents with a mental illness with focus on eating disorders

**DOI:** 10.1186/s12888-022-04349-5

**Published:** 2022-11-16

**Authors:** Adéla Farářová, Hana Papežová, Jana Gricová, Tereza Štěpánková, Václav Čapek, Charlotte Reedtz, Camilla Lauritzen, Karin van Doesum

**Affiliations:** 1 First Faculty of Medicine, Charles University, Prague, Czech Republic; 2 Department of Psychiatry, First Faculty of Medicine, Charles University and General University Hospital in Prague, Prague, Czech Republic; 3 RKBU North, Faculty of Health, UiT – Arctic University of Norway, Tromsø, Norway; 4 Department of Clinical Psychology, Radboud University, Nijmegen, Netherlands

**Keywords:** ChildTalks+, COPMI, Family prevention intervention, Child mental health prevention, Risk factors, Protective factors, Eating disorders

## Abstract

**Background:**

Children of parents with a mental illness are at high risk of developing a mental disorder as a result of transgenerational transmission. Without effective intervention, they could form the next generation of psychiatric patients. ChildTalks+ is a preventive intervention involving four structured psychoeducational sessions designed for parents affected by a mental disorder and their children. Its aim is to reduce the risk of mental disorders in children of parents with mental illness. This study draws on our clinical practice and involves a group of patients with eating disorders. The aim of the project, which will run in the Czech Republic, is to evaluate the effectiveness of ChildTalks+ methodology.

**Methods:**

ChildTalks+ therapists (professionals from health, social, and educational facilities) will recruit 66 families where a parent is treated for a mental disorder and the family includes children aged 6–18. Paired allocation into an intervention group (*N* = 33) and a control group (*N* = 33) will be based on the number of risk factors identified in the family. Both groups will complete questionnaires at the baseline, post-test, and follow-up assessments after six and 12 months. The intervention group will receive the ChildTalks+ intervention within 2 months of the baseline assessment; the control group after the last assessment. Questionnaires will be completed by parents and children aged 12+ and, in two cases, 15+ years. Quantitative data will be supplemented with qualitative data from ChildTalks+ therapists working with patients with eating disorders.

**Discussion:**

The ChildTalks+ intervention is expected to strengthen parenting competencies and family protective factors, improve family communication, increase awareness of parental mental health issues, and improve the wellbeing of children of parents with mental illness with long-term sustainable outcomes. The study should contribute to the evidence base for the ChildTalks+ program and help identify key themes in the implementation of similar preventive interventions.

**Trial registration:**

ClinicalTrials.gov Identifier: NCT05554458. Registered 26 September 2022. Retrospectively registered.

## Background

Parental mental disorder is a significant biological and environmental risk factor, which affects app. 15–23% of young people who live with parents with a mental disorder worldwide. Children whose parents are affected by a mental disorder (COPMI) have up to a 50% chance of developing a mental illness themselves [1]. They are 5.2 times more likely to develop depression and 3.7 times more likely to develop anxiety disorders than their peers [2]. Given these data on transgenerational transmission of disorders and lack of research on the effectiveness of preventive interventions, COPMI may represent the next generation of patients with mental disorders [3–5]. Although in recent years, this high-risk group has received more attention, provision of assistance to COPMI has not yet become a clear public health policy priority.

### Transgenerational transmission and risk factors

The paradigm of transgenerational transmission of risks leading to mental disorders in offspring posits five transmission mechanisms: genetic and prenatal influences, parent–child interactions, family processes and conditions, and social influences outside the family [6]. It must, however, be considered that mental disorders in parents have different levels of genetic component and present themselves by different symptoms and effects on parental behavior. As a consequence, they also affect the offspring in various ways [5]. Transmitted psychopathology of parents can lead in their offspring to similar but also different mental disorders. The concept of equifinality refers to a single disorder or problem in COPMI that is the result of different risk trajectories and exposure to various types of parental diagnoses. In contrast, the concept of multifinality denotes the impact of parent’s particular disorder or specific risk factor on the offspring which manifests itself in the form of various disorders or social outcomes [3, 5, 7].

Regardless of parent’s diagnosis, COPMI face in consequence of presence of a mental disorder in their family similar risks. These general, nonspecific risks include increased and prolonged stress, feelings of loneliness, guilt and shame, poor academic performance, identity problems, difficulties in establishing intimate relationships, and increased risk of suicidal behavior, as well as parental neglect, abuse, and maltreatment [3, 6, 8–11]. In a similar fashion, parents with mental disorders and their offspring are at risk of poverty and conflicts within the family or their neighbors [3, 6]. Specific risks associated with particular diagnostic groups of mental disorders also play a role. Parental modeling in the form of pathological coping strategies – such as externalizing behavior, substance abuse, or emotional overeating – can lead to reinforcement of specific pathologies in the offspring [3, 6].

### Protective factors

Having mentioned the role of both nonspecific and specific risk factors, we should also highlight the role of protective factors, which increase resilience, prevent the development of psychopathology in the COPMI, and buffer the impact of risk factors. For the most part, they are not specific to the parental disorder [6]. Protective factors include a safe bond between the child and parents, the care of the other parent without a mental disorder, the child’s personality, temperament, and available coping strategies, as well as social support within the family and wider social network [3, 7, 12, 13]. To achieve positive outcomes and greater effectiveness of preventive interventions, a comprehensive approach to COPMI consider specific and nonspecific risks as well as protective factors [3, 6, 7].

### The theoretical model

**Fig. 1:**
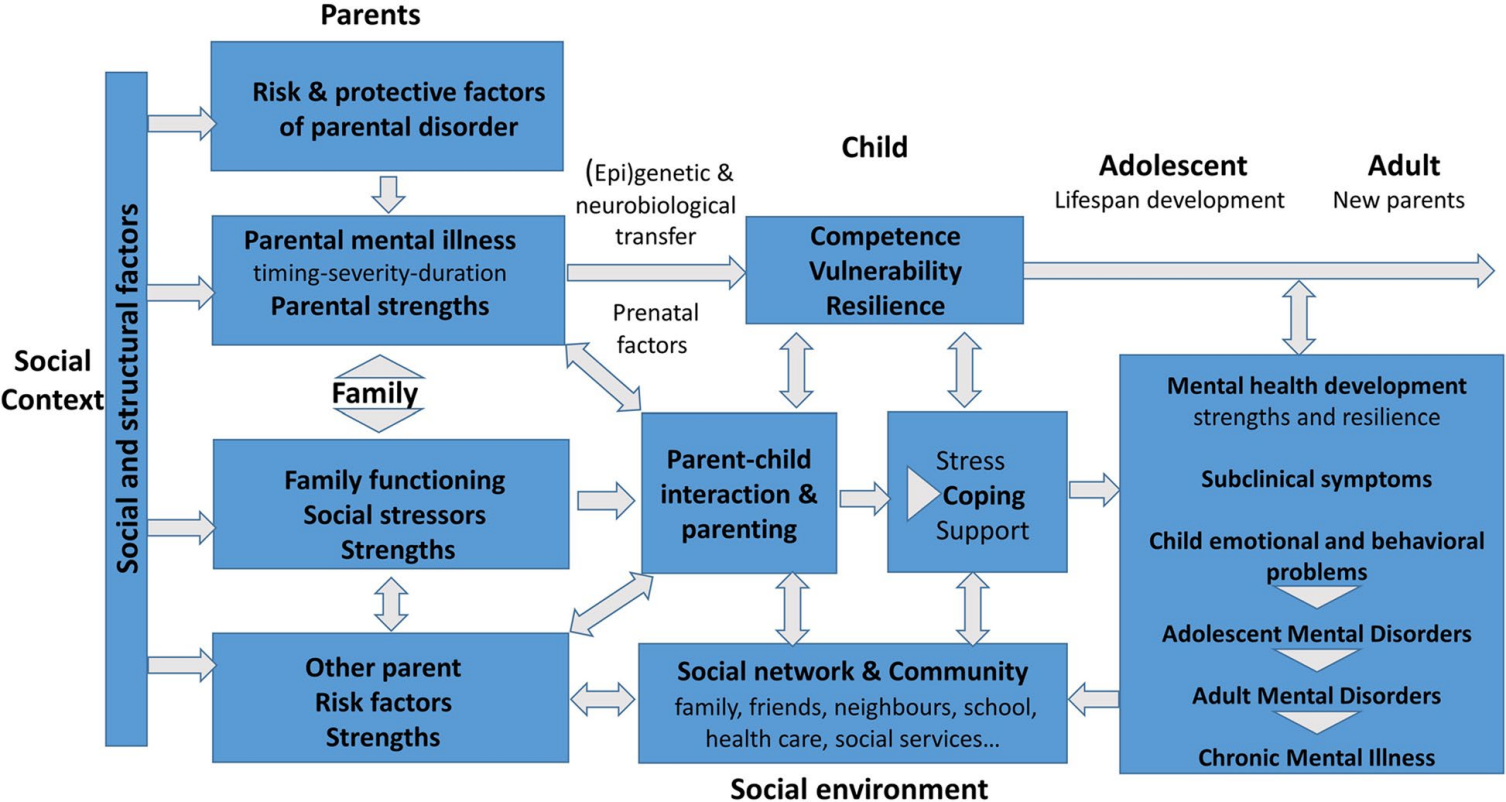
The theoretical model

This theoretical model of transmission mechanisms and factors in families with parental mental disorders proposed by Hosman & Van Doesum [3, 6, 14] (see Fig. 1, printed with the authors’ permission) describes the main domains of risk factors and protective factors in the development of mental health and psychopathology in COMPI. In our study, paired allocation of families will be based on selected risk factors that influence transgenerational psychopathology.

### Preventive interventions

Several previous studies investigated the effectiveness of interventions aimed at families affected by mental disorder of a parent [4, 15, 16]. One of the first systematic reviews and meta-analyses of interventions reported a significant (40%) relative reduction (seven studies) of risk of children developing the same disorder as their parent or parents, with only a small overall effect for children internalizing the symptoms and a nonsignificant effect for externalizing symptoms of the parent’s disease [16]. A study that investigated severe mental disorders in parents and community-based interventions found small, nonsignificant, and merely short-term effects on children’s emotional health and social functioning. Medium to large size effects were observed for parents’ depressive symptoms and parenting behavior [15]. A meta-analysis of 96 articles, including 50 independent samples from randomized controlled trials, reported that interventions targeting parents and children together had a larger overall effect [4]. It has also been reported that involvement of parents in preventive interventions increased their awareness of potential harmful effects of their mental illness on their children and improved emotional support and family bonds. Moreover, it aided the implementation of preventive measures, because children are more easily accessible through their parents [14, 17–20].

Clinical practice in adult mental healthcare therefore ought to move toward adopting a family-centered approach, identify the vulnerable COPMI, and support them through preventive interventions provided by mental health professionals [1, 3, 14, 21–26]. While some parents are supportive of such efforts, others are reluctant to participate because they fear that it might affect their childcare or even lead to a loss of custody of their children. They may tend to shield their children from information about their mental disorder [14]. In either case, creation of a system of stable collaboration between professionals in adult mental health care and professionals in pediatric and adolescent mental health care may support parents with a mental disorder and their offspring and facilitate their engagement in preventive interventions [26–28].

### Psychoeducation

Psychoeducation in particular has proven to be an indispensable part of prevention, useful for both parents and children [4, 16, 19, 29]. Along with other common components (skills training, training of emotion regulation and play activities in the family, as well as multifamily and group interventions, etc.), it is in 87.5% of cases a key component of targeted psychological treatment programs for COPMI [30]. Psychoeducation provides an opportunity to learn appropriate strategies for coping with the stress and crises that occur in the family. Consequently, it contributes to the establishment and strengthening of positive bonds within families which may be negatively affected by a parent’s mental disorder [31, 32]. COPMI who receive through psychoeducation accurate and non-stigmatizing information about their parent’s mental illness, treatment, and recovery may reach a better understanding of their parents’ behavior, talk about the situation with others, and feel less alone [10, 33–35]. Children appreciate psychoeducation provided by mental health professionals [36, 37]. The messages they receive can reduce potential experienced stigma, outline hope for a recovery from mental illness, and reassure them that the parent’s disorder is not their fault [35].

### The current study

Given that, to the best of our knowledge, there is in the Czech Republic no methodology for a targeted and systematic prevention of future mental health problems in children whose parents suffer from mental illness, we decided to implement and evaluate the ChildTalks+, which is a family-focused intervention. This methodology has the advantage of considering the specific ranges of difficulties resulting from particular mental disorders. Moreover, its authors are active in aiding this methodology’s implementation in several countries by training and other assistance.

### Implementation issues

Identification and support of COPMI through adult mental health care can be challenging and this large population of high-risk children tends to go unnoticed [26]. Given the risk to COPMI, their care ought to be supported by legislative changes. In the Netherlands, where the ChildTalks+ intervention was among the first to be introduced, all mental health services currently offer preventive interventions for children whose parents have a mental illness and their families [6]. In Norway, health legislation introduced in 2010 made it mandatory to identify whether patients with mental health disorders have children and to offer these families adequate support. In the northern region of Norway, the ChildTalks+ intervention has been implemented as the instrument that would provide the intervention mandated by this legislation. Results show that this legislative change had assisted a better identification of COPMI, but a decade after its introduction neither support nor follow-up on these vulnerable individuals are a routine part of counsellors’ skills [7, 38, 39].

### Parents with eating disorders

Within the pilot project Implementation and evaluation of ChildTalks+ in the Czech Republic, we decided to include parents with eating disorders in addition to parents with various other diagnosed mental disorders. Our interest in the former group was codetermined by our clinical practice at the Centre for Diagnosis and Treatment of Eating Disorders at the Psychiatric Clinic of the First Faculty of Medicine of the Charles University and General University Hospital (both in Prague, Czech Republic). Moreover, the number of people with eating disorders is increasing [40, 41] and over 70% of these individuals have comorbidities [41], mainly anxiety and depression [42]. These disorders, which are associated with high mortality [43], are among diagnostic groups with a high risk of transgenerational transmission of psychopathology [44], although factors that affect it are subject of further research [44–48]. Eating disorders are classified as hereditary disorders influenced by both genetic and environmental factors [45]. Advanced genetic research continues to identify differences in exposure to environmental risk factors [46–48] but, in general, findings pertaining to transgenerational transmission of eating disorders suggest that the process if driven by multiple risk mechanisms [42, 49–52]. Given that women are at a higher risk of eating disorders than men, majority of research has been focused on maternal eating disorders and their effect on cognitive and psychological development of the offspring [46, 52, 53].

### Untreated parent as a risk factor for the offspring

Alarmingly, a significant number of adults, including parents, receive no treatment [45], and only a third of adult patients with eating disorders are detected by the healthcare system [41]. Individuals do not sign up for treatment for many reasons, including a feeling of shame associated with having an eating disorder and a desire to keep it a secret from children [50, 54]. Mothers who describe the perceived impact of the disorder on their children and their relationship with them report a sense of failure in the parenting role, a fear that they are not good role models for their children, but also helplessness and despair at their inability to cope with the disorder and humiliation when the disorder manifests itself in the home environment [50, 54].

Given the abovementioned increasing trend in the prevalence of eating disorders, low utilization of the healthcare system, and the high risks which children of patients with eating disorders face [42], introduction of effective prevention and screening programs for this group are much needed [46, 48, 55]. Since eating disorders are also associated with concealment of illness [50, 56], it is important to assess the feasibility of prevention programs in the light of possible reluctance of parents with eating disorders to participate and to consider factors that could motivate their participation. Further research on the risks, resilience, and protective factors influencing the development of eating disorders in the offspring will reveal further information pertinent to the deployment and targeting of these factors in the prevention and treatment of eating disorders [47, 48, 57–62]. This is an area to which, we hope, our study could contribute.

### Aims of the study

The aim of this study is to evaluate the effectiveness of the ChildTalks+ intervention and to implement the intervention in training and in practice. Our aim is to deliver the ChildTalks+ intervention, that is, educate parents about the transgenerational transmission of their disorder, inform them about its impact on their children, strengthen their parenting competencies, support communication within the family, but also inform the children about their parent’s mental disorder, listen to their needs, and provide emotional and social support to the family. This, we hope, should lead to improved family communication (including children’s awareness of their parents’ mental health problems), improved overall wellbeing of COPMI, increased perceived parental competence, and strengthening of family protective factors, including strengthened social support, sustained over time. Part of the intervention consists of early identification of social and emotional problems in COPMI and referral for further professional help.

Our research questions are chiefly the following:

- What are the effects of the ChildTalks+ intervention in families where a parent has a mental health disorder?
- Is the ChildTalks+ intervention feasible for therapists who treat patients with mental disorders?
- Is the ChildTalks+ intervention feasible in families where one parent has an eating disorder?
- Should the ChildTalks+ intervention be modified for families where a parent has an eating disorder?

## Methods/design

### Trial design

**Fig. 2:**
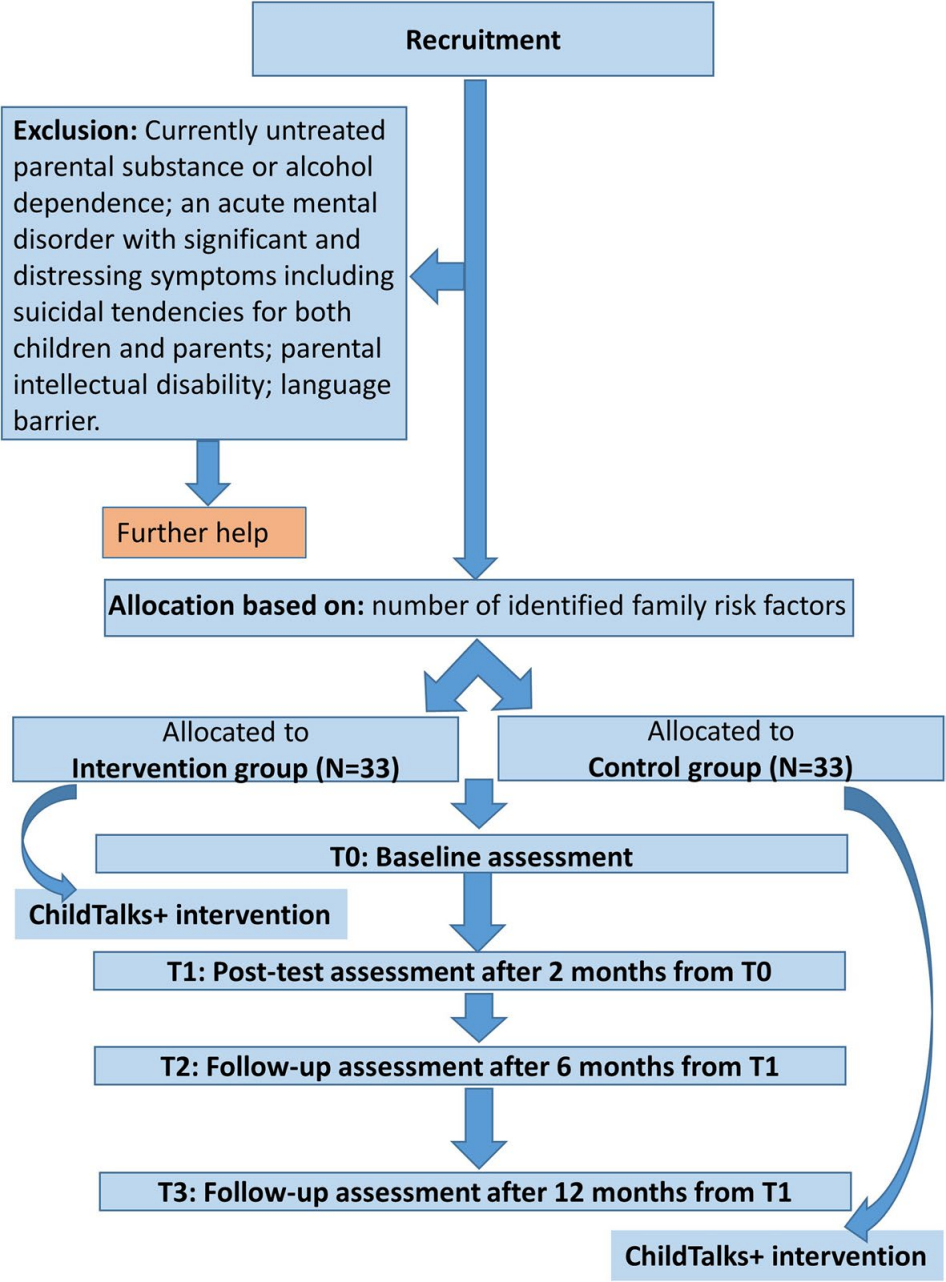
The study design

The current study is designed as quasi-experimental, pre–post controlled, paired design, with two conditions: an intervention group and a control group. Planning of the study protocol drew on the Model Protocol by the Arctic University of Norway [28]. Figure 2 shows the study design, including the recruitment, inclusion and exclusion criteria, group allocation, and assessments at baseline, post-test, and follow-up, as well as the measures used.

### Study settings

Implementation and evaluation of the ChildTalks+ intervention will take place in the Czech Republic. Participation will be offered to professionals from healthcare, social, and educational institutions (especially pedagogical–psychological counselling centers), who will become ChildTalks+ therapists after completing the training. Trained therapists will then recruit families with children aged 6–18, where one or both parents have been formally diagnosed with a mental illness. In addition to a wide range of mental disorders, our clinical specialization will help us focus on families where a parent has been diagnosed with an eating disorder. The recruitment of respondents will run in three waves. New ChildTalks+ therapists will be trained in each wave.

### Participants

Families with children aged 6–18, where one parent or both have a formal diagnosis of a mental disorder, will be recruited by ChildTalks+ therapists, that is, professionals who work in healthcare, social care, or education. In the case a relevant family includes several children aged 6–18, it is recommended that the study should include all of them.

### Eligibility criteria

Families will be eligible for inclusion according to the following criteria: at least one parent is treated for a mental disorder (according to DSM-5 or ICD-10 diagnostic criteria) and at least one child in the family is aged 6–18.

Exclusion criteria for families: parental substance or alcohol dependence that is currently untreated; an acute mental disorder with significant and distressing symptoms including suicidal tendencies that requires an immediate treatment for both children and parents; parental inability to provide consent due to intellectual disability; language barrier.

Eligibility criteria for therapists implementing the ChildTalks+ intervention: professional competence to work with families; ability to adapt the content and language of sessions to the type of parent’s disorder and the age group of the child (ren) involved; completion of training in the ChildTalks+ intervention; motivation to actively participate in the project.

### Procedure

Therapists will be recruited by a therapist coordinator in collaboration with the project coordinator. Once trained, ChildTalks+ therapists will begin working with families. They will first give the potential candidates information about the ChildTalks+ intervention and about participation in the study. This research study is an essential part of project Implementation and Evaluation of ChildTalks+ in the Czech Republic. If potential candidates express interest, they will be asked if they agree that their other family members be contacted, because our aim is to involve entire families. Participation will, however, be also possible for pairs consisting of just one parent with a mental health disorder and one child aged 6–18. For children aged under 18, parental consent is required for their participation in the study. Participants will receive a timeline regarding the survey measures and delivery of the ChildTalks+ intervention. They will receive reminders of upcoming meetings.

Families will pay nothing for their participation in the project and receive no financial reward. Trained therapists will be financially rewarded for their work with families: the remuneration will cover a total of 10 h of work with each family. This includes direct work with the family using the ChildTalks+ methodology and administration of questionnaires (handing them out, providing instructions for completion, collection of completed questionnaires, and their delivery to coordinators of the implementation team).

### The ChildTalks+ intervention

ChildTalks+ is a preventive intervention originally developed in the Netherlands, which targets children up to 18 years of age. It has been implemented in Norway, Italy, and Portugal. It has a clear and well-described theoretical foundation focused on psychoeducation. Its key aim is to provide feasible and replicable interventions to improve the quality of life of families where one or both parents are affected by a mental disorder [6, 7, 28, 63, 64]. As noted above, this intervention has broad applicability: it is suitable for children and adolescents whose parents have any psychiatric diagnosis, as well as for COPMI from families with specific problems in certain diagnostic groups. This intervention moreover facilitates early detection and identification of subclinical and clinical forms of mental disorders in COPMI and referral for further support [26, 28, 63, 64].

The intervention takes the form of four psychoeducational sessions, two with parents only and two with the whole family, including children. During the sessions, a trained therapist talks with the family about its situation. The aim is to improve the parents’ awareness of possible impact of the disorder on the child and to support them in their parenting competences. Subsequently, the therapist provides the child with age-appropriate information about the parent’s disorder, treatment, and recovery, as well as with emotional and social support, thereby strengthening the child’s ability to cope with mental disorders. This encourages communication within the family [28, 63, 64]. Replicability of the ChildTalks+ methodology makes it accessible to a wide range of mental health professionals who, once trained, can effectively disseminate it to the target population in a variety of settings, thus improving access to prevention for more COPMI [26, 28, 63, 64].

Several studies [26, 63] described the implementation of the Child Talks intervention, which is a modification of the ChildTalks+ intervention consisting of just three sessions, but no studies have as yet assessed the effectiveness of the ChildTalks+ intervention [63]. A study conducted in Norway collected and evaluated electronic patient diaries written by mental health professionals. It reported that children participating in the Child Talks intervention were more likely to know about their parents’ health status because they lived with a parent who had been hospitalized. The study has also identified certain key themes for COPMI: communication about the parent’s mental disorder and the child’s situation, and the need for additional support from mental health professionals [63]. A study conducted in Portugal focused on changing the clinical practice regarding the identification of COPMI by mental health professionals. Introduction of the Semente program, which is based on the Child Talks intervention, confirmed significant changes in clinical practice in the pre- and post-measurement period. The greatest changes were observed in improved provision of a family-centered approach and in the clarity and access to policies and procedures among professional staff [26].

### Description of the ChildTalks+ meetings

#### Meeting 1

A therapist trained in the ChildTalks+ intervention conducts an initial meeting, if possible, with both parents/primary caregivers. If this is not possible, only the parent with mental health difficulties may be present. At the start, the therapist explains the purpose of the meeting and encourages the parents to view the mental disorder from the child’s perspective. The therapist talks to them about possible impact of the mental disorder on their children and family life and discusses with the parents possible protective factors for the family.

#### Meeting 2

Both parents should attend also the second meeting. The purpose of this meeting is to give parents advice and guidance on how to discuss the mental disorder at home. Different approaches can be taken for instance, a role play. At the end of the second meeting, the therapist and parents should prepare for the next meeting, in which the children will participate. Parents should indicate whether they prefer to conduct the conversation with their children themselves or whether they would prefer the therapist to do it. They work on the central themes that will require the most attention.

#### Meeting 3

Children should attend the third meeting together with their parents. One of the main goals of this session is to find out how the children are coping with the situation. The focus is on the children’s own experience of relationships with parents and peers. The parent or the therapist answer the children’s questions. The therapist tries to gain insight into the children’s coping strategies, strengths, and resilience. During this session, children should receive emotional support and sufficient, age-appropriate information about the parent’s mental disorder.

#### Meeting 4

The fourth and final meeting is held, if possible, with both parents and their children. Its purpose is to summarize and evaluate the previous meetings. The therapist answers the family’s questions and allows parents and children to express their perspectives. All this should help parents gain a better understanding of their children’s needs and to be better equipped to meet them. Subsequent steps are discussed with the family, including options for follow-up care [28, 64].

**Fig. 3:**
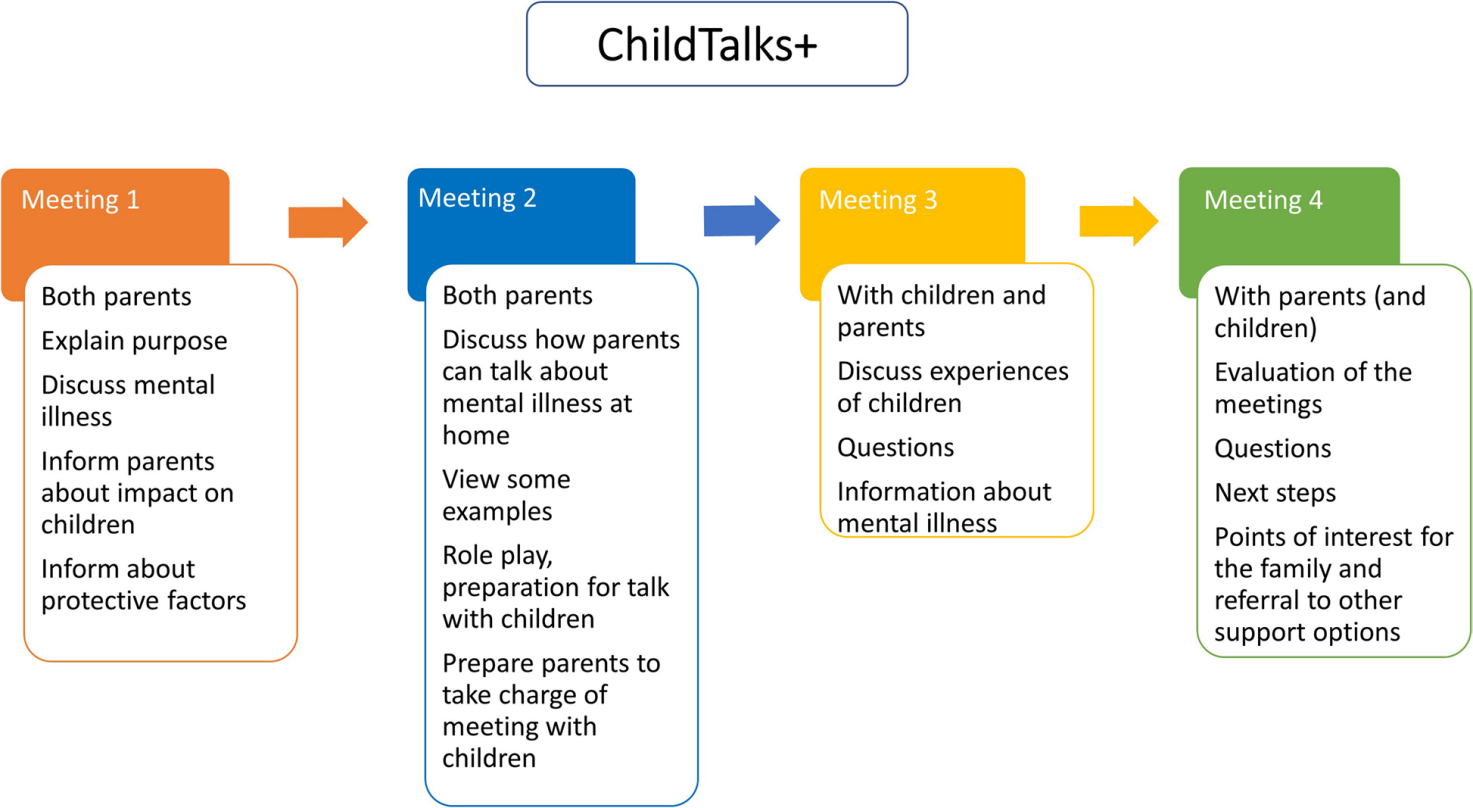
Overview of the Child Talks+ meetings

Figure 3 presents an overview of the Child Talks+ meetings [64]. The authors’ permission to publish has been obtained.

### Intervention integrity

The manualization, training, and supervision carried out as part of ChildTalks+ should help ensure consistency and quality of program delivery. The training of therapists will take 2 days, follow clearly defined procedures, and be led by authors of the ChildTalks+ intervention. Trained therapists will have access to the manualized ChildTalks+ intervention procedures, including brochures on mental disorders for parents and children and information about particular mental disorders. Trained therapists will follow the intervention manual and complete standard checklists (logbook) for each session.

### Allocation into groups

Using a quasi-experimental design, each family will be assigned to a condition (intervention or control group) based on a paired matching. Allocation to a group will be based on the number of identified risk factors in the family [3]. These include mental disorder/comorbidities of the respondent, frequency of hospitalizations, diagnosis of a mental disorder of the other parent, eventually the child or children, absence of another caregiver, ignorance of the respondent’s diagnosis within the family, and low household income. Common demographic data (gender, age, highest educational attainment, the number of adults and children in the household) are also collected from respondents.

### Sample size and power calculation

Power analysis was computed based on the Model Protocol by Reedtz et al [28] We hypothesized that the within-subject change of the outcome between T0 and T1 should differ by about 2.5 between the intervention and the control group, with a pooled SD of the scores app. 4.90. For the power analysis, a simple two-tailed paired t-test with significance level of 5% was used to get a power of 0.8 with an estimated sample size of 33 pairs. Trained therapists will offer participation to 80–88 families, thus allowing for some attrition. A total of 66 families will be assigned to the intervention (*N* = 33) or control (*N* = 33) group.

### Statistical methods

The null hypothesis, according to which the within-subject change of the outcome between T0 and T1 will be the same for the intervention and the control group against a two-sided alternative (immediate effect), will be tested using a linear mixed-effects model with two nested random effects, where the paired families will be first level no. 1 and the family the second level no. 1. The model will be adjusted for factors which are likely to affect the outcome. In the same manner, we will assess the null hypothesis about the within-subject change of the outcome between T0 and T2 (medium term effect) and between T0 and T3 (long term effect). *P*-values will be adjusted for multiple comparisons using the Holm’s method. Analyses will be performed using the R Statistical Software.

### Assessments

The data will be collected using a variety of questionnaires (described under measures). The questionnaires are completed by parents of children aged 6–18 and children aged 12 and older. Two questionnaires on the list, namely *The Strengths and Difficulties Questionnaire (SDQ)* and *The Youth Mental Health Literacy Scale (YMHL)*, will be completed by children over the age of 15. For children under 12 years of age, the Ethics Committee of the General University Hospital (Prague, Czech Republic) allowed qualitative data to be collected through interviews with the therapist. Participating parents and children will explore areas which are being assessed at the baseline, post-test (T1), and at the follow-ups (T2, T3). All assessments will be conducted using appropriate scales individually with each subject.

#### The intervention group (*N* = 33)

After the baseline assessment (T0), recruited families should receive the ChildTalks+ intervention. Two months after T0, assessment will be repeated in a post-test (T1). Parents and children should also receive a follow-up assessment 6 months from T1 (at T2) and 12 months from T1 (at T3).

#### Control group (*N* = 33)

Two months after the baseline assessment (T0), post-test assessment (T1) should be performed at the same time as the T1 assessment of the intervention group. Parents and children have a follow-up assessment 6 months from T1 (T2) and 12 months from T1 (T3). After the T3 follow-up assessment, families will receive the ChildTalks+ intervention.

In the qualitative part of the research, we also explore the use of the ChildTalks+ intervention by professionals who treat patients diagnosed with eating disorders. We will gather these qualitative data using semi-structured interviews to explore the feasibility of ChildTalks+ intervention for patients with eating disorders and their offspring, and to describe the therapists’ experiences during the collaboration, including perceived obstacles and positives. The results should also inform us about what parents and children found helpful about the ChildTalks+ intervention. The qualitative data will be assessed by a thematic analysis [65].

### Measures

*Health-Related Quality of Life (KIDSCREEN-27)* [66, 67] is a measure used to assess children’s health-related quality of life, a factor that is receiving ever more attention and is considered an important indicator of health status in both pediatric and epidemiological research. The scale is designed for children aged 8–18. It contains 27 questions divided into five subscales on how well the child feels physically and mentally, on perceived autonomy and relationships with parents, social support and relationships with peers, and the school environment. All items use the same five-point Likert scale. Scores are reported as t-values, with higher scores reflecting a higher health-related quality of life. The scale takes 10–15 minutes to complete [28].

*Parent–Child Communication Scale* [68] consists of two scales: the child report and the parent report. The Parent–Child Communication Scale assesses how children and their primary caregivers perceive each other’s openness to communication and their communication skills. The child report consists of 10 items, the parent report of 20 items. Responses are coded on a five-point Likert scale ranging from 1 (“almost never”) to 5 (“almost always”) [28].

*Parenting Sense of Competence (PSOC)* [69, 70] is a scale containing 17 questions for parents who have a child or children aged 6–18. There is a version of the questionnaire adapted for mothers and for fathers. The aim of the scale is to assess how accurately parents perceive their ability to do the job of parenting their children. The scale contains factors that reflect satisfaction and efficiency, where satisfaction reflects parents’ motivation, anxiety, and frustration, while effectiveness reflects parenting competence, including problem-solving skills. Subscales are rated on a 6-point scale from 1 (“strongly agree”) to 6 (“strongly disagree”). This measure does not differentiate based on the children’s age or gender. It has been demonstrated that this scale has an adequate reliability for use with parents [28].

*Parental Evaluation of Developmental Status (PEDS)* [71, 72] is a scale designed for parents of children aged from 21 months to 8 years. It consists of 10 questions. In answering them, parents describe their concerns regarding significant emotional or behavioral problems related to their children’s development. If parents’ concerns are carefully identified, they can help detect mental health difficulties in the child’s development. This scale enables a categorizarion of children into risk groups for developmental disorders based on different types of parental concerns. In other words, the measure helps determine whether children are at a high, medium, limited, or no risk of developmental problems. Previous studies have established that the measure’s reliability scores are adequate [28].

*Eating Questionnaire – Youth version (ChEDE-Q)* [73, 74] contains 39 questions validated for children aged 6–18. The questions focus on the child’s eating habits, relationship with own body, and physical activity. Children answer them according to the reality of the past 28 days. By analysing the answers, one can obtain information about the child’s risk of developing eating disorders.

*The Strengths and Difficulties Questionnaire (SDQ)* [75, 76] is a scale used to assess the strenghts and weaknesses of children aged 6–18. The Ethics Committee of the General University Hospital in Prague had decided that it can only be administered to children over 15 years of age. This screening questionnaire consists of 25 questions divided in five areas: emotional symptoms, conduct problems, hyperactivity and inattention, interpersonal problems, and prosocial behavior. This questionnaire is completed separately by parents and children. Responses to the statements are rated on a three-point Likert scale (“not true,” “somewhat true,” “certainly true”). According to the prevalence of clinical disorders, children with higher total difficulty scores show greater psychopathology. Previous studies have shown that this scale’s reliability scores are adequate, and the measure is considered a dimensional tool for assessing children’s mental health [28].

*Youth Mental Health Literacy Scale (YMHL)* [33] scale examines children’s awareness of mental health (common mental illnesses/holistic recovery), mental illness stigma, coping and help-seeking for self or other people who may be experiencing what could be mental health symptoms. Children’s knowledge of mental health and recovery measures can be used for the general population and/or the COPMI [33]. The scale was originally developed for children aged 11–16. For the purpose of the pilot study in the Czech Republic, the measure’s authors agreed that it could be administered to children aged 11–18 who would be divided in two groups (11–14 years and 15–18 years). But the Ethics Committee of the General University Hospital in Prague had decided that it can only be administered to children over 15 years of age.

## Discussion

Given the prevalence and burden of transgenerational transmission of mental disorders in COPMI, it is imperative that effective evidence-based prevention interventions be identified and disseminated as a routine part of care for patients with mental illness who are parents and in care of their children. The vulnerable COPMI group struggles with lack of parents’ awareness of mental health issues and the parents are often not aware of the child’s perspective, i.e., the child’s experience of the situation. Issues of stigma can prevent parents from having relevant conversations. For children, it is important understand their parents’ behavior in relation to their mental disorder, while parents need to understand the impact of mental illness on their child’s behavior. Parents and children welcome having information relayed to them by a mental health professional. In the long term, we expect that the ChildTalks+ intervention will reduce emotional problems in COPMI and prevent (further) mental health problems from developing.

The aim of this study will be to introduce and pilot test the preventive ChildTalks+ intervention for children whose parents suffer from a mental disorder. The results should demonstrate the efficacy of the ChildTalks+ methodology, including its sustainability over time, and thereby contribute new knowledge regarding the risk and protective factors acting on COPMI. The results should also objectively describe feasibility of this prevention program that is based on working with children through their parents. Given our specialization on patients with eating disorders, we will address the effects and feasibility of the ChildTalks+ intervention especially with respect to children whose parents have eating disorders.

### Strengths and limitations

One of the strengths of this study is that the program will be based on full adherence to the ChildTalks+ methodology, including training of therapists by authors of the program. We will assess the efficacy of the program in a trial with nearly 70 families where a parent has a mental illness and the family includes children aged 6–18. The study design encourages participation by the entire family, that is, including the partners of persons with mental illness and all children in the family. Potential positive results from the study would support a broader use of the ChildTalks+ methodology in preventive mental health services not only in the Czech Republic but also in other countries. An additional strength is the involvement of mental health professionals as ChildTalks+ therapists. For therapists who work with patients with eating disorders, accessibility and feasibility of the intervention will be documented through additional qualitative data, which should provide information regarding the awareness of risk and protective factors for the disorder. Finally, to avoid high attrition rates, parents and children will be fully informed about requirements related to the study-related prior to enrolment. Therapists will assist parents and children with completing the questionnaires.

A limitation of this study is that parents may find it difficult to talk about their mental health problems because of fears of stigma. Fear of admitting the true extent of their problems may lead to reluctance regarding either their own or their children’s participation in the study. Another limitation is that many parents with mental health problems who are experiencing symptoms of illness have not been diagnosed by a relevant professional and therefore cannot be identified. Finally, there is a limitation related to the age of the children: based on the decision of the Ethics Committee, children younger than 12 or 15 will not be able to complete the questionnaires needed to evaluate the study.

### Implication for practice

If implemented in clinical practice, the ChildTalks+ program could be a significant preventive intervention aimed at helping the COPMI. Treatment centers should expand their focus to the entire family, not care for adults in isolation from care for their offspring. Parents struggling with a mental disorder usually do not communicate with their children about their condition and related difficulties. Breaking this silence may improve the parent–child interaction and children may be able to better understand their own as well as their parents’ situation. This may increase the competence of both parents and children, leading to a better quality of life regardless of the severity of parent’s mental disorder.

## Data Availability

Data from projects using this study protocol should be made available to researchers through approved data repositories in accordance with the relevant legislation. The results of this study will be made available to the scientific community through peer-reviewed publications in scientific journals. It is intended that results of this study would be published internationally, presented at conferences, and disseminated through international research groups. Policymakers, public health and social services, and stakeholders will be informed via conferences, websites, newsletters, and personal contacts.

## Abbreviations

COPMI: Children of Parents with Mental Illness
IG: Intervention group
CG: Control group
